# Nano-scale Al redistribution at grain boundaries governs growth morphology in β-(Al_x_Ga_1-x_)_2_O_3_ on sapphire substrate via MOCVD

**DOI:** 10.1186/s11671-026-04753-w

**Published:** 2026-07-06

**Authors:** Chih Yang Huang, Filip Gucmann, Anoop Kumar Singh, Po-Hsun Chen, Chien-Nan Hsiao, Edmund Dobročka, Milan Ťapajna, Igor Píš, Matej Mičušík, Siddharth Rana, Dong-Sing Wuu, Po-Liang Liu, Kenneth Järrendahl, Ching-Lien Hsiao, Ray-Hua Horng

**Affiliations:** 1 Institute of Electronics, National Yang Ming Chiao Tung University, Hsinchu, 30010 Taiwan; 2 Institute of Electrical Engineering, Slovak Academy of Sciences, Dúbravská Cesta 9, 841 04 Bratislava, Slovakia; 3 Department of Applied Materials and Optoelectronic Engineering, National Chi Nan University, Nantou, 54561 Taiwan; 4 Graduate Institute of Precision Engineering, National Chung Hsing University, Taichung, 40227 Taiwan; 5 National Center for Instrumentation Research, National Institutes of Applied Research, Hsinchu, 300092 Taiwan; 6 Thin Film Physics Division, Department of Physics, Chemistry and Biology (IFM), Linköping University, 581 83 Linköping, Sweden; 7 Electronic and Optoelectronic System Research Lab., Industry Technology Research Institute, Hsinchu, 31041 Taiwan; 8 Polymer Institute, Slovak Academy of Sciences, Dúbravská Cesta 9, 845 41 Bratislava, Slovakia; 9 Institute of Pioneer Semiconductor Innovation, National Yang Ming Chiao Tung University, Hsinchu, 30010 Taiwan

**Keywords:** Ultra-wide-bandgap, β-(Al_x_Ga_1−x_)_2_O_3_, MOCVD, Atomic-resolution STEM, First-principles

## Abstract

Ultra-wide-bandgap β-Ga_2_O_3_ is a promising platform for next-generation deep-UV optoelectronics and high-power electronics. Here, we report controlled growth of β-(Al_x_Ga_1−x_)_2_O_3_ epilayers on sapphire by metalorganic chemical vapor deposition with tunable trimethylaluminum supply (2.24 × 10^−6^–3.36 × 10^−5^ mol min^−1^). Systematic X-ray diffraction peak shifts toward higher angles confirm progressive Al incorporation and lattice contraction, while cross-sectional microscopy reveals an increase in film thickness from 153 to 278 nm, indicating enhanced nucleation and growth kinetics. Optical spectroscopy demonstrates tunable bandgaps from 4.92 to 5.66 eV, and Vegard’s-law analysis quantifies Al contents of ~ 6–36%. Atomic-resolution STEM combined with first-principles calculations reveals nano-scale Al redistribution and demonstrates that Al incorporation significantly lowers the surface energy of the O-terminated (100) facet, promoting lateral growth and yielding characteristic rectangular platelet morphologies. These results establish a quantitative thermodynamic origin for morphology control in β-(Al_x_Ga_1−x_)_2_O_3_ and provide a framework for morphology-engineered ultra-wide-bandgap oxide electronics.

## Introduction

β-Ga_2_O_3_, offering an ultra-wide bandgap of about 4.8 eV, has become a focal material for emerging power and optoelectronic applications [1–6]. In contrast to conventional wide-bandgap materials, β-Ga_2_O_3_ offers the advantage of being readily grown by multiple scalable and economically viable methods, including mist-CVD, pulsed laser deposition, metal–organic chemical vapor deposition (MOCVD), hydride vapor phase epitaxy, and magnetron sputtering [7–13]. Owing to its strong breakdown strength of approximately 8 MV/cm and superior Baliga’s figure of merit, β-Ga_2_O_3_ positions ahead of Si, GaAs, SiC, and GaN shows promise in enhanced power device performance [14]. Recent advances have highlighted the importance of bandgap engineering through the formation of (Al_*x*_Ga_1-*x*_)_2_O_3_ alloys, especially for (Al_*x*_Ga_1-*x*_)_2_O_3_/β-Ga_2_O_3_ interfaces [15, 16]. Incorporation of Al_2_O_3_ into the Ga_2_O_3_ epilayer enables a tunable bandgap reaching up to ~ 8.8 eV, which broadens its potential in deep-ultraviolet photodetectors and high-voltage applications requiring even higher critical field strengths [17]. Furthermore, the existence of a two-dimensional electron gas (2DEG) at (Al_*x*_Ga_1-*x*_)_2_O_3_/β-Ga_2_O_3_ interfaces facilitates the development of high-frequency, high-power modulation-doped field effect transistors (MODFETs) [18–20]. Earlier studies have demonstrated a 2DEG sheet charge density of ~ 2 × 10^12^ cm^−2^ in β-(Al_*x*_Ga_1-*x*_)_2_O_3_/β-Ga_2_O_3_ MODFETs, with mobility up to 2790 cm^2^/V s [18]. However, increasing Al content can lead to significant structural transitions from the β-phase toward metastable κ- or γ-Al_2_O_3_ phases [21–24], which alter the electronic band structure, carrier transport, and defect characteristics. Thus, a detailed insight of the interplay amidst alloy composition, crystal structure, and electronic properties is essential. MOCVD is widely regarded as a scalable technique for producing high-quality epitaxial layers with precise control over precursor supply [25, 26]. For β-(Al_x_Ga_1-x_)_2_O_3_, MOCVD provides a tunable pathway to tailor composition and film thickness through simple adjustments to growth temperature, pressure, and flow rate of precursors [27–29]. Despite extensive interest in β-(Al_x_Ga_1-x_)_2_O_3_ for ultrawide-bandgap optoelectronics, a comprehensive understanding of how Al precursor simultaneously impacts growth kinetics, structural evolution, and bandgap tuning remains limited. Prior reports have largely focused on either compositional control or optical characterization, with fewer studies systematically correlating precursor chemistry, thickness evolution, and surface thermodynamics [30, 31]. In particular, accurate determination of Al content by utilizing the precursor is lacking, which limits the ability to correlate composition with physical properties. In this work, we extensively examine the growth mechanism, as well as the structural and electronic characteristics of (Al_*x*_Ga_1-*x*_)_2_O_3_ epilayers deposited on sapphire substrates under varying TMAl molar flow rates (2.24 × 10^–6^ to 3.36 × 10^–5^ mol/min). The Al composition is rigorously quantified using precise measurement of interplanar spacing ${d}_{\left(\overline{2 }01\right)}$ by XRD and calculations assuming its linear change with the varied Al content, i.e., Vegard’s law. Optical bandgap measurements are employed to validate the composition-dependent electronic properties. This work offers essential insights into the structure–property relationships of (Al_x_Ga_1-x_)_2_O_3_ ternary alloys and provides a framework for the design of ultra-wide bandgap devices with tailored functionalities.

## Experimental

β-Ga_2_O_3_ epilayers incorporating varying Al compositions were deposited on 2-inch *c*-plane sapphire (Al_2_O_3_) substrates employing MOCVD. Trimethylaluminum (TMAl), triethylgallium (TEGa), and oxygen (O_2_) served as the Al, Ga, and O precursors, respectively. The growth process was conducted at a temperature of 825 °C and a chamber pressure of 50 torr. The TEGa flow rate was fixed at 3.65 × 10^–5^ mol/min, while the TMAl flow rate was systematically varied from 2.24 × 10^–6^ to 3.36 × 10^–5^ mol/min to control the Al concentration in the (Al_*x*_Ga_1-*x*_)_2_O_3_ alloys. The total growth time was 60 min.

The crystallinity of the resulting (Al_*x*_Ga_1-*x*_)_2_O_3_ epilayers was characterized using an X-ray diffraction (XRD) system (Malvern PANalytical Aeris Benchtop). The Al molar fraction (x) in the (Al_*x*_Ga_1-*x*_)_2_O_3_ alloys was determined using a diffractometer (Bruker D8 DISCOVER), featuring a 12 kW rotating Cu anode. The measurements employed a parallel beam geometry, incorporating a parabolic Göbel mirror in the incident path. Symmetrical 2θ/ω scans were performed over a 2θ range of 15°–80°. To minimize strong diffraction signals from the sapphire substrate, each sample was tilted by 0.5° relative to the substrate’s precise diffraction position.

A JEOL JSM-7610F field-emission scanning electron microscope (FE-SEM) was utilized to determine the thickness and surface morphology of the films. The microstructural properties were analyzed with a 200 kV STEM (FEI Titan ChemiSTEM) embedded with an aberration corrector, providing a beam current, probe size, and convergence semi-angle of 70 pA, 1 Å, and 19.8 mrad, respectively. High-angle annular dark-field (HAADF) STEM imaging were acquired with a detector angular range spanning 7.9–42.8 mrad at atomic resolution. The optical bandgaps of the epilayers were estimated from normal incidence transmittance spectra measured in the 190–1000 nm wavelength range (n&k 1280, n&k Technology Inc.).

To evaluate the Al concentration (*x*) in the (Al_*x*_Ga_1-*x*_)_2_O_3_ alloys by the XRD measurement, we assumed that increasing Al content causes the continuous change of the β-Ga_2_O_3_ lattice parameters $A$, $B$, $C$ and $\beta$ towards the parameters of monoclinic Al_2_O_3_ (JCPDS PDF 00-023-1009), i.e., and isomorphic counterparts of β-Ga_2_O_3_. To determine the (Al_*x*_Ga_1-*x*_)_2_O_3_ lattice parameters, at least five independent diffraction measurements are needed. Consequently, the Al content can be calculated assuming a linear dependence of the lattice parameters. The precision of the Al content evaluation can be further improved by increasing the number of measurements.

A more straightforward approach to determine the Al content ($x$) is to focus on a single lattice plane. If the variations of lattice parameters $A\left(x\right)$, $B\left(x\right)$, $C\left(x\right)$, and angle $\beta \left(x\right)$ with composition are nearly linear, this assumption can be applied to all interplanar spacings. The ($\overline{2 }01$) plane, which is parallel to the sample surface, is particularly suitable for this analysis. Using a standard symmetrical scan, three reflections, ($\overline{2 }01$), ($\overline{4 }02$), and ($\overline{6 }03$), can be measured. Based on Vegard’s law, the interplanar spacing ${d}_{\left(\overline{2 }01\right)}$ is assumed to vary linearly with Al content $x$:

1 $$ d_{{\left( {\bar{2}01} \right)}} \left( x \right) = d_{{\left( {\bar{2}01} \right)}}^{{{\mathrm{GaO}}}} \left( {1 - x} \right) + d_{{\left( {\bar{2}01} \right)}}^{{{\mathrm{AlO}}}} x, $$

2 $$ x = \frac{{d_{{\left( {\bar{2}01} \right)}}^{{{\mathrm{GaO}}}} - d_{{\left( {\bar{2}01} \right)}} }}{{d_{{\left( {\bar{2}01} \right)}}^{{{\mathrm{GaO}}}} - d_{{\left( {\bar{2}01} \right)}}^{{{\mathrm{AlO}}}} }}. $$

where ${d}_{\left(\overline{2 }01\right)}^{\mathrm{G}\mathrm{a}\mathrm{O}}$ and ${d}_{\left(\overline{2 }01\right)}^{\mathrm{A}\mathrm{l}\mathrm{O}}$ are the interplanar spacings of pure β-Ga_2_O_3_ and Al_2_O_3_ phases, respectively. A standard reference value (${d}_{\left(\overline{2 }01\right)}^{\mathrm{A}\mathrm{l}\mathrm{O}}=0.454 \mathrm{n}\mathrm{m}$) and measured value of ${d}_{\left(\overline{2 }01\right)}^{\mathrm{G}\mathrm{a}\mathrm{O}}$ was used in the calculations for minimal errors. This method helps to minimize the impact of lateral strain in the layer and reduces the effects caused by the experimental conditions. The accuracy of the parameter mainly relies on the difference in interplanar spacing between the β-Ga_2_O_3_ and Al_2_O_3_ phases. By differentiating expression (2), a relationship is established between the uncertainties ($\delta x$,$\delta {d}_{\left(\overline{2 }01\right)}$)and in the following form:

3 $$ \delta x = \frac{{\delta d_{{\left( {\bar{2}01} \right)}} }}{{d_{{\left( {\bar{2}01} \right)}}^{{{\mathrm{GaO}}}} - d_{{\left( {\bar{2}01} \right)}}^{{{\mathrm{AlO}}}} }}. $$

Thus, a smaller difference between interplanar spacing of β-Ga_2_O_3_ and Al_2_O_3_ (${d}_{\left(\overline{2 }01\right)}^{\mathrm{G}\mathrm{a}\mathrm{O}}-{d}_{\left(\overline{2 }01\right)}^{\mathrm{A}\mathrm{l}\mathrm{O}}$) results in higher uncertainty when determining the value of $x$ in (Al_*x*_Ga_1-*x*_)_2_O_3_.

X-ray photoelectron spectroscopy (XPS) signals were recorded using a Thermo Scientific Nexsa G2 Surface Analysis System (Thermo Fisher Scientific, UK) equipped with a micro-focused, monochromatic Al Kα X-ray source (8.34 Å,1486.68 eV). An X-ray beam of 400 µm size was used. The spectra were acquired in the constant analyzer energy mode with pass energy of 200 eV for the survey. Narrow regions were collected using the pass energy of 50 eV. Charge compensation was achieved with the system dual beam flood gun. Ion etching was done using 40 s of ion gun sputtering (2 keV Ar^+^ ions with a high current density over an area of 8 mm^2^). The Thermo Scientific *Avantage* software, version 6.10.0 (Thermo Fisher Scientific), was used for digital acquisition. Spectral calibration was determined by using the automated calibration routine and the internal Au, Ag and Cu standards supplied with the NEXSA G2 system.

The surface compositions (in atomic%) were determined by considering the integrated peak areas of the detected atoms and the respective sensitivity factors. The fractional concentration of a particular element A was computed using:

4 $$ \% ~A~ = ~\frac{{I_{A} /s_{A} }}{{\sum \left( {I_{n} /s_{n} } \right)}}~ \times 100\% , $$

where *In* and *sn* are the integrated peak areas and the relative sensitivity factors (RSF) corrected for the analyzer transmission, respectively. The RSFs were estimated by multiplying the theoretical photoionization cross section [32] by the inelastic mean free path, as determined by the TPP-2 M [33] formula for Ga_2_O_3_. A slight preferential sputtering of oxygen was corrected by analyzing the peak areas before and after ion etching.

In addition to experimental characterization, first-principles simulations were conducted to elucidate the surface energetics of β-Ga_2_O_3_ and Al-incorporated (Al_*x*_Ga_1-*x*_)_2_O_3_ surfaces. The bulk β-Ga_2_O_3_ unit cell was constructed using the monoclinic *C*2/*m* crystal structure and contains 20 atoms: 8 Ga and 12 O. The Perdew–Wang 1991 (PW91) exchange–correlation functional within the generalized gradient approximation (GGA) was employed in the Vienna *Ab-initio* Simulation Package (VASP) [34–38]. The energy cut-off was set to 450 eV, and *k*-point sampling was performed using a 2 × 8 × 4 Gamma-centered grid. After structural optimization, the calculated lattice parameters were *a* = 12.44 Å, *b* = 3.10 Å, *c* = 5.89 Å, and β = 103.70°, which are in good agreement with previously reported theoretical and experimental results for β-Ga_2_O_3_ [39]. The β-Ga_2_O_3_ (100) surface model was constructed with an O-terminated Ga_2_O_3_(100) surface and cleaved to contain 42 atoms (16 Ga and 26 O). Additionally, a 75 Å vacuum layer was introduced. Geometry optimization was conducted using the same energy cut-off of 450 eV and a *k*-point grid of 6 × 3 × 1. To model Al incorporation, an Al atom was then substituted for one Ga atom in the slab to generate the O-terminated Ga_15_Al_1_O_26_(100) slab model, which was similarly optimized under the same computational parameters. This modified structure was also optimized under identical computational conditions. These simulations aimed to elucidate the influence of Al incorporation on surface stability and the morphology evolution observed experimentally.

To further quantify the thermodynamic driving force for Al-induced lateral growth, a β-Ga_2_O_3_ grain boundary model was constructed using the open-source Aimsgb toolkit. The framework is based on the Coincidence Site Lattice (CSL) theory, which generates symmetric tilt grain boundaries when two grains are rotated by specific misorientation angles that allow partial coincidence of lattice points at the interface. Following the procedure developed by Cheng et al. [40] and the segregation analysis of hydrogen in aluminum grain boundaries by Afzalimir et al. [41], an initial β-Ga_2_O_3_ Σ5(201)[010] symmetric tilt grain-boundary structure was generated. To ensure physically meaningful bonding, all interatomic distances and bond topologies were screened and corrected. Atoms separated by distances smaller than 1.0 Å and homonuclear bonds (e.g., O–O or Ga–Ga) were removed to preserve the ionic-bonding characteristics of the oxide system. After the CSL-based grain-boundary construction, atomic relaxation and total-energy minimization were performed to eliminate artificial local strain and unrealistic bonding configurations introduced during the initial geometrical setup. The Σ5(201)[010] configuration was selected as a representative low-Σ, symmetric-tilt grain boundary within the CSL framework to investigate Al segregation behavior near the β-Ga_2_O_3_ grain boundary. Previous high-throughput DFT grain-boundary studies have shown that low-Σ symmetric tilt grain boundaries are commonly used as representative grain-boundary models owing to their relatively high structural coherence, lower interface energies, and the high computational cost associated with first-principles grain-boundary calculations [42]. In addition, recent studies on β-Ga_2_O_3_ have demonstrated that highly coherent grain boundaries stabilized by local pseudomirror symmetry can exhibit low interface energies and play important roles in defect formation and epitaxial growth behavior [43].

**Fig. 1:**
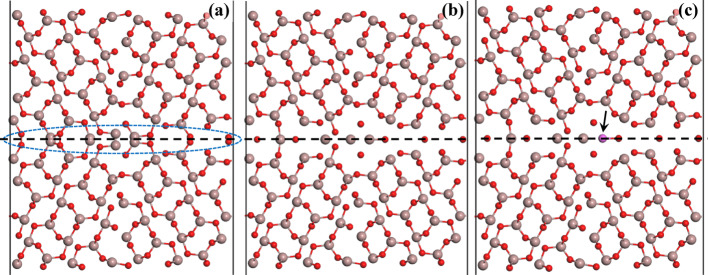
Structural models of the β-Ga_2_O_3_ Σ5(201)[010] grain boundary used for segregation energy calculations. Gray, red, and pink spheres represent Ga, O, and Al atoms, respectively. **a** Initial grain-boundary structure generated according to the CSL theory, where the deep-blue rectangle highlights unphysical atomic configurations with excessively short interatomic distances (< 1.0 Å) or homonuclear bonds (O–O and Ga–Ga) arising from the geometric coincidence construction. **b** Corrected grain-boundary model after removal of unrealistic bonds and local structural relaxation, resulting in a physically reasonable β-Ga_2_O_3_ Σ5(201)[010] interface consistent with the ionic-bonding nature of the oxide. **c** Al-doped grain-boundary structure in which one Ga atom at the boundary plane is substituted by an Al atom; the blue dashed line marks the Σ5 boundary plane. The arrow indicates the position of the Al atom at the Σ5 grain boundary

As illustrated in Fig. 1, the construction sequence involves three key steps: generation of the raw CSL boundary, elimination of geometrically inconsistent atomic pairs, and targeted substitution of a single Al atom at the interface. The corrected model ensures that all bond lengths fall within the typical Ga–O ionic-bond range (1.8–2.0 Å), thereby eliminating spurious high-energy bonds prior to first-principles relaxation. The refined model contained 135 Ga atoms and 202 O atoms, and was subsequently relaxed until the total energy converged, thereby eliminating artificial strain introduced during geometric generation. This approach yields a realistic grain boundary with coordination and bonding characteristics representative of β-Ga_2_O_3_. To evaluate the energetic preference of Al incorporation at the grain boundary, the segregation energy was calculated using:

5 $$ E_{{{\mathrm{seg}}\left( {{\mathrm{Al}}} \right)}} = E_{{{\mathrm{GB}} + {\mathrm{Al}}}} {-}E_{{{\mathrm{GB}}}} - \, (E_{{{\mathrm{bulk}},{\mathrm{Al}}}} - E_{{{\mathrm{bulk}}}} ), $$

where *E*_GB+Al_ and *E*_GB_ are the total energies of the Al-doped and pristine grain boundary models, respectively, while *E*_bulk,Al_ and *E*_bulk_ denote the total energies of Al-doped and pristine bulk β-Ga_2_O_3_. A negative *E*_seg(Al)_ indicates that replacing Ga by Al at the boundary is energetically favorable, implying a thermodynamic driving force for Al segregation toward the interface.

## Results and discussion

**Fig. 2:**
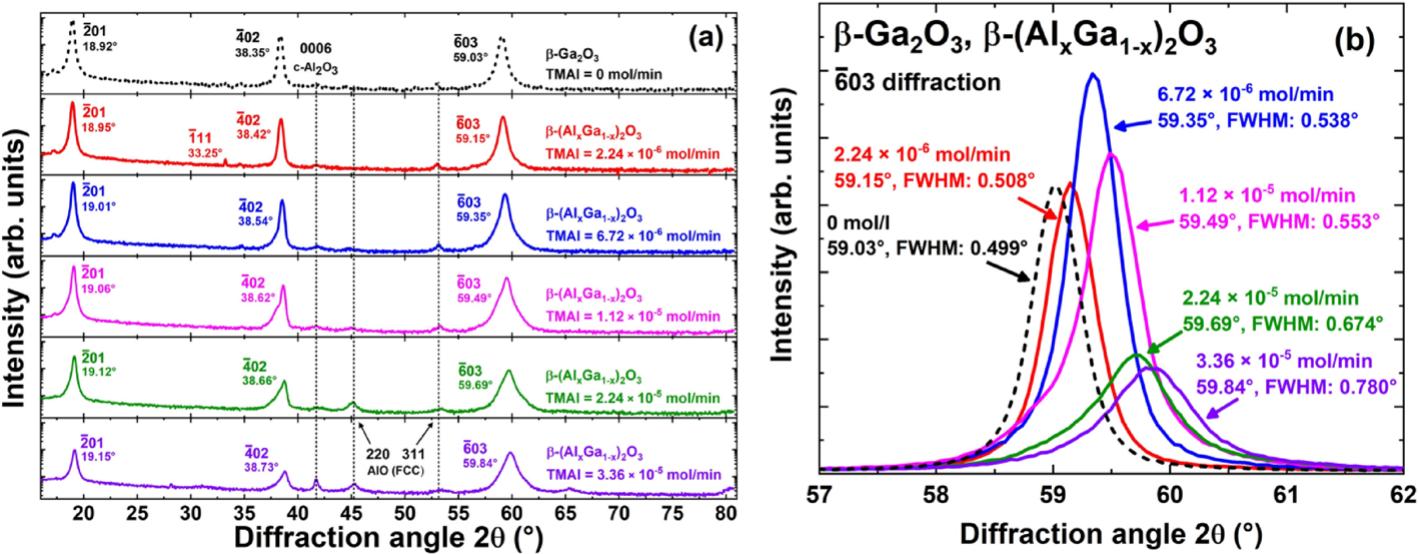
**a** Wide-angle symmetric XRD patterns, and **b** $\overline{6 }$03 diffraction peaks of (Al_x_Ga_1−x_)_2_O_3_ epilayers with varying molar flow rates of TMAl

The XRD patterns of (Al_*x*_Ga_1-*x*_)_2_O_3_ epilayers, demonstrating structural evolution of deposited epilayers via MOCVD with different TMAl molar flow rates (~ 2.24 × 10^–6^ to ~ 3.36 × 10^–5^ mol/min), are presented in Fig. 2. Three dominant diffraction peaks $\overline{2 }$01, $\overline{4 }$02, and $\overline{6 }$03 corresponding to the ($\overline{2 }$01) lattice reflections, similar to monoclinic β-Ga_2_O_3_ [44, 45] were observed, which indicated that the monoclinic phase dominated the epilayer structures. Here,** t**he diffraction peak of sapphire substrate served as a benchmark. The effect of the molar flow rate of TMAl on the crystallinity is also significant in the XRD patterns, causing the intensities of the three primary diffraction $\overline{2 }$01, $\overline{4 }$02, and $\overline{6 }$03 to decrease. Nevertheless, the presence of the structure similar to β-Ga_2_O_3_ remains unchanged. It was also found that as the TMAl molar flow rate increased, these peaks shifted toward higher diffraction angles. When comparing pristine β-Ga_2_O_3_ with Al-rich samples (grown with a TMAl molar flow rate of 3.36 × 10^–5^ mol/min) the $\overline{2 }$ 01 diffraction peak shifted from 18.90° to 19.14°, the $\overline{4 }$02 diffraction peak shifted from 38.33° to 38.78°, and the $\overline{6 }$03 diffraction peak shifted from 58.96° to 59.82°. It is suggested that this is caused by a lattice parameter reduction due to the substitution of larger Ga^3+^ ions (~ 0.62 Å) with smaller Al^3+^ ions (~ 0.54 Å) for. This shift confirms the inclusion of Al into the (Al_*x*_Ga_1-*x*_)_2_O_3_ epilayers. Figure 2b shows a detail of the XRD with the $\overline{6 }$03 diffraction peak for (Al_*x*_Ga_1-*x*_)_2_O_3_ epilayers deposited with varying molar flow rates of TMAl, in comparison with pristine β-Ga_2_O_3_. The full width at half maximum (FWHM) of the $\overline{6 }$03 diffraction peaks for pristine β-Ga_2_O_3_ and (Al_*x*_Ga_1-*x*_)_2_O_3_ epilayers grown with TMAl molar flow rates of 2.24 × 10^–6^, 6.74 × 10^–6^, 1.12 × 10^–5^, 2.24 × 10^–5^, and 3.36 × 10^–5^ mol/min were measured to be 0.499°, 0.508°, 0.538°, 0.553°, 0.674°, and 0.780°, respectively. The FWHM initially increases only slightly, indicating minimal distortion at low Al incorporation. This effect may be ascribed to the partial substitution of Ga^3+^ ions with the smaller Al^3+^ ions, which may help to fill lattice vacancies and reduce local structural disorder. The variation in the FWHM of the X-ray diffraction peaks indicates changes in the structural properties of the films. Although Al substitution can reduce certain point defects, it also introduces lattice distortion due to Al ions, having a reduced radius than Ga ions. The lattice mismatch with c-sapphire varies slightly with Al content [46]. However, the overall FWHM trend results from the competing effects of reduced point defects and strain-induced broadening.

We note, that decreasing grain size and Al content fluctuations can also contribute to increased FWHM values of evaluated diffraction peaks. Additional reflections were present in (Al_x_Ga_1-x_)_2_O_3_ X-ray diffractograms. The peak at ~ 41.7° correspond to 0006 reflection of sapphire substrate; only small contribution of this reflection is visible, as the samples were deliberately tilted away from the exact substrate diffraction by an angle of 0.5° to achieve its suppression and allow the (Al_x_Ga_1-x_)_2_O_3_ reflections to resolve clearer. Peaks at ~ 45° and ~ 53° are most likely related to cubic Al_2_O_3_ (PDF 01-075-0278), and correspond to 220 diffraction of (110)-oriented grains and to reflection from (311) plane, respectively. Other minor peaks, present predominantly in the film with the highest Al content are attributed to increased crystal disorder. We note that these low intensity peaks do not agree with expected diffractions from any Ga_2_O_3_ phase and are therefore attributed to localized structural variations rather than the formation of a distinct secondary phase, indicating no notable significant phase separation occurred in the grown films. The increased intensities of the 220 and 311 peaks at higher TMAl molar flow rates suggest enhanced local structural inhomogeneity and possible onset of localized phase segregation.

The measured diffraction patterns were evaluated by the TOPAS 3.0 software. The obtained interplanar spacings ${d}_{\left(\overline{2 }01\right)}$, the associated uncertainty $(\delta d$), and the calculated values of the Al molar fraction $x$ are summarized in Table 1 for varied TMAl molar flow rates. The value ${d}_{\left(\overline{2 }01\right)}^{\mathrm{G}\mathrm{a}\mathrm{O}}=0.46903 \mathrm{n}\mathrm{m}$ of the Al free pristine Ga_2_O_3_ sample was used in the calculation.

**Table 1:** XRD-determined interplanar spacings ${d}_{\overline{2}01 }$, associated uncertainty $\delta d$, and calculated Al molar fraction *x* of (Al_x_Ga_1−x_)_2_O_3_ films for varied molar flow rates of TMAl

TMAl molar flow rate [mol/min]	${d}_{\overline{2}01 }$[nm]	$\delta d$[nm]	$x$[-]
0	0.46903	0.00003	0.00
2.24 × 10^−6^	0.46812	0.00002	0.06
6.72 × 10^−6^	0.46691	0.00002	0.14
1.12 × 10^−5^	0.46587	0.00003	0.21
2.24 × 10^−5^	0.46448	0.00004	0.30
3.36 × 10^−5^	0.46356	0.00003	0.36

According to Eq. (3), the uncertainty of interplanar spacing δ*d* ~ 0.00004 nm is transferred to the uncertainty of Al content δ*x* ~ 0.003. Other sources contributing to the uncertainty may include some degree of ${d}_{\overline{2}01 }(x)$ deviation from linearity and the uncertainty of the tabulated value of ${d}_{\left(\overline{2 }01\right)}^{\mathrm{A}\mathrm{l}\mathrm{O}}$. It is therefore reasonable to estimate the upper limit of $\delta x$ as 0.01 (~ 1%).

**Fig. 3:**
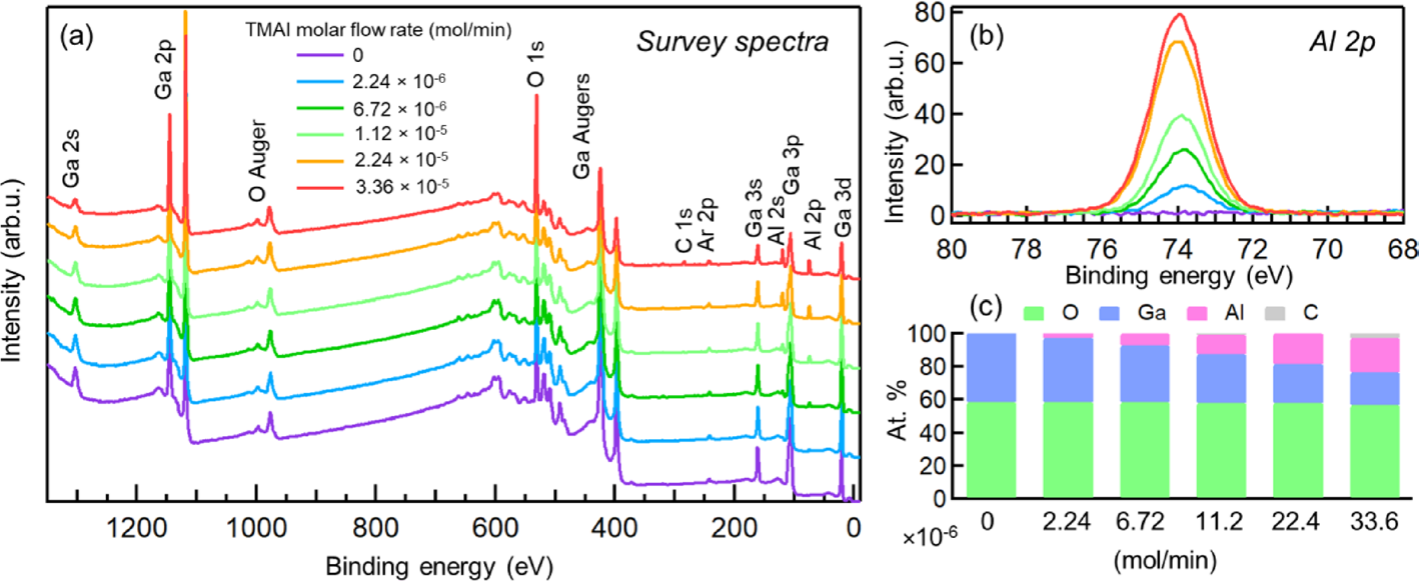
XPS results for (Al_x_Ga_1−x_)_2_O_3_ epilayers with varying molar flow rates of TMAl. **a** Survey spectra, **b** detail of Al 2p signals and** c** bar chart with atomic concentrations of O, Ga, Al, and C

XPS was used to evaluate the Al molar fraction in (Al_x_Ga_1-x_)_2_O_3_ films and to support the Al-content evaluation by the XRD. Along with the Al, atomic concentrations of O, Ga, and C were evaluated. Determined values are summarized in Table 2. Figure 3a shows survey spectra for all the investigated samples grown using TMAl molar flow rates from 0 to 3.36 × 10^–5^ mol/min with description of the main observed signals. Figure 3b details the Al 2p signal used to determine the Al content and Fig. 3c presents a bar chart with atomic concentration of O, Ga, Al, and C visualizing their individual contributions.

**Table 2:** XPS-determined atomic concentrations of O, Ga, Al, and C and calculated Al molar fraction *x* of (Al_x_Ga_1−x_)_2_O_3_ films for varied molar flow rates of TMAl

TMAl molar flow rate [mol/min]	Atomic concentrations [%]				$x$[-]
	O	Ga	Al	C	
0	57.9	42.1	0.0	0.0	0.00
2.24 × 10^−6^	57.9	38.9	2.9	0.3	0.07
6.72 × 10^−6^	57.8	34.3	7.3	0.0	0.19
1.12 × 10^−5^	57.2	29.5	11.8	1.5	0.29
2.24 × 10^−5^	57.3	23.4	18.6	0.7	0.44
3.36 × 10^−5^	56.2	19.6	20.9	3.3	0.52

Good agreement, within the experimental uncertainty, was found between XRD- and XPS-determined Al molar fractions in the investigated (Al_*x*_Ga_1-*x*_)_2_O_3_ epilayers for films grown with TMAl molar flow rates from 0 to 1.12 × 10^−5^ mol/min. In contrast, notably higher Al molar fraction was found by XPS for film grown with TMAl molar flow rate of 2.24 × 10^−5^ mol/min (0.44 vs 0.30) and for film grown with TMAl molar flow rate of 3.36 × 10^−5^ mol/min (0.52 vs 0.36). The elevated Al content detected by XPS may be associated with the formation of aluminum oxide, which was also indicated by the XRD analysis. Since XPS measurement is surface-sensitive technique, we expect the discrepancy results from higher Al-content near the films surface. As reported elsewhere, high-Al content in monoclinic (Al_*x*_Ga_1-*x*_)_2_O_3_ films can cause phase separation when Al solubility limit is reached [47, 48], however the solubility limit may differ significantly with the chosen growth conditions and used substrate material. We observed slight O1s signal broadening for high Al-contents (not shown) which can indicate differences in Al and Ga bonding and an onset of phase separation, which may be co-responsible for the observed differences. It is to be noted that XPS is a surface-sensitive technique probing only the near-surface region, while XRD provides bulk-averaged structural information. Therefore, the higher Al composition determined by XPS at higher TMAl flow rates may also originate from compositional nonuniformity through the film thickness, particularly Al enrichment near the surface region.

**Fig. 4:**
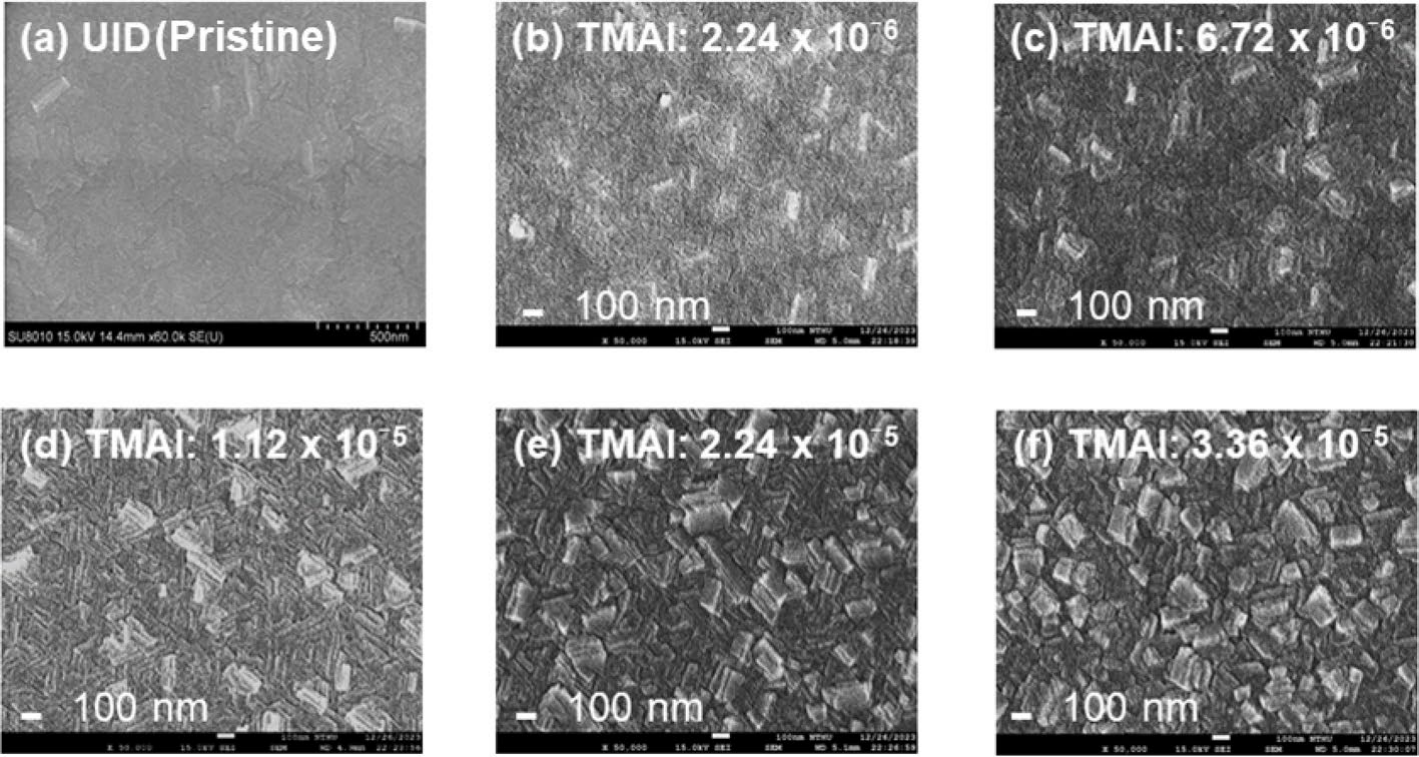
Top-view SEM micrographs of **a** pristine β-Ga_2_O_3_ and (Al_*x*_Ga_1-*x*_)_2_O_3_ epilayers grown with **b** 2.24 × 10^−6^ mol/min, **c** 6.72 × 10^−6^ mol/min, **d** 1.12 × 10^–5^ mol/min, **e** 2.24 × 10^–5^ mol/min, and **f** 3.36 × 10^–5^ mol/min TMAl molar flow rate (white scale bar represents 100 nm in all micrographs)

The surface features of the (Al_*x*_Ga_1-*x*_)_2_O_3_ epilayers were examined using FESEM, as presented in Fig. 4. The pristine β-Ga_2_O_3_ and the sample grown with a low TMAl molar flow rate of 2.24 × 10^–6^ mol/min exhibited relatively smooth surfaces with less defined grain boundaries shown in Fig. 4a and b. This suggests limited surface diffusion and low Al incorporation. That is, the morphology did not change much and maintained the same morphology as that of pristine β-Ga_2_O_3_.

The surface morphology gradually developed well-defined facets with increasing molar flow rate of TMAl. At 6.72 × 10^–6^ mol/min (shown in Fig. 4c), faceted grains began to emerge, indicating the onset of enhanced lateral growth. With further increases in TMAl flow (Fig. 4d–f), the surface evolved to feature more compact and regularly shaped grains with distinct crystal facets and well-defined grain boundaries. Notably, the sample grown at 3.36 × 10^–5^ mol/min (Fig. 4f) displayed prominent faceted crystallites and the most developed grain structure among all samples.

These observations indicate that higher Al incorporation enhances adatom mobility and surface crystallization, thereby creating more grains, which degrades the epilayer’s crystallinity [49]. The increased grain size and faceting suggest a transition toward a more energetically favorable growth mode with elevated Al content, consistent with the structural changes seen in the XRD. This correlation highlights the significant role of Al doping on the microstructural evolution of (Al_*x*_Ga_1-*x*_)_2_O_3_ epilayers.

**Fig. 5:**
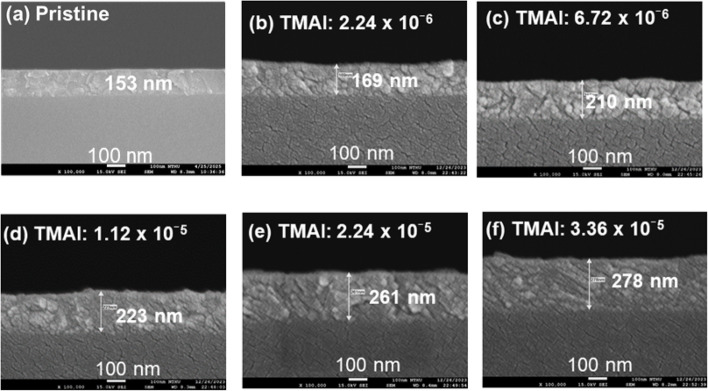
Cross-sectional SEM micrographs of (Al_*x*_Ga_1-*x*_)_2_O_3_ epilayers deposited with varied molar flows of TMAl: **a** 0 (pristine Ga_2_O_3_), **b** 2.24 × 10^–6^ mol/min, **c** 6.72 × 10^–6^ mol/min, **d** 1.12 × 10^–5^ mol/min, **e** 2.24 × 10^–5^ mol/min, and **f** 3.36 ×10^–5^ mol/min

Figure 5a–f shows cross-sectional SEM micrographs of (Al_*x*_Ga_1-*x*_)_2_O_3_ epilayers deposited with various molar flow rates of TMAl. The thickness of the (Al_*x*_Ga_1-*x*_)_2_O_3_ epilayers is measured to be 153 nm, 169 nm, 210 nm, 223 nm, 261 nm, and 278 nm for pristine β-Ga_2_O_3_, and epilayers with 2.24 × 10^–6^ ~ 3.36 × 10^–5^ mol/min TMAl molar flow rates, respectively. An enhanced growth rate for higher TMAl molar flow rate is indicated. These results clearly demonstrate a positive correlation between the epilayer thicknesses and TMAl molar flow rate. The pristine β-Ga_2_O_3_ epilayer exhibits a thickness of 153 nm, while Al-doped samples grown for 60 min exhibit increasing thickness with higher TMAl molar flow rate, reaching a maximum of 278 nm at 3.36 × 10^–5^ mol/min. The increase in epilayer thickness with increasing TMAl molar flow rate is consistent with the monotonic high-angle shift of the XRD peaks, indicating systematic modification of the cell parameters due to Al incorporation. These observations suggest that TMAl molar flow rate influences both the structural evolution and growth kinetics of the films, potentially by increasing the density of nucleation sites or altering surface energy dynamics.

**Fig. 6:**
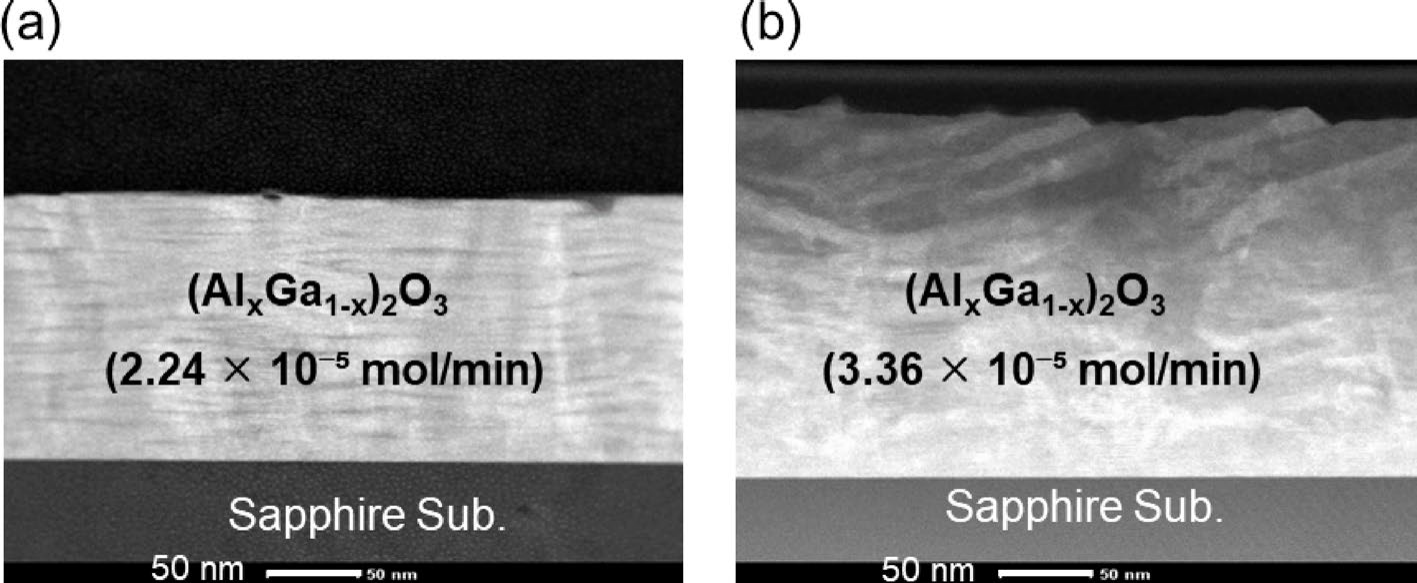
STEM images with a low magnification obtained from the MOCVD deposited β-(Al_*x*_Ga_1-*x*_)_2_O_3_ thin films with different molar flow rates of TMAl: **a** 2.24 × 10^–5^ mol/min, and **b** 3.36 × 10^–5^ mol/min

**Fig. 7:**
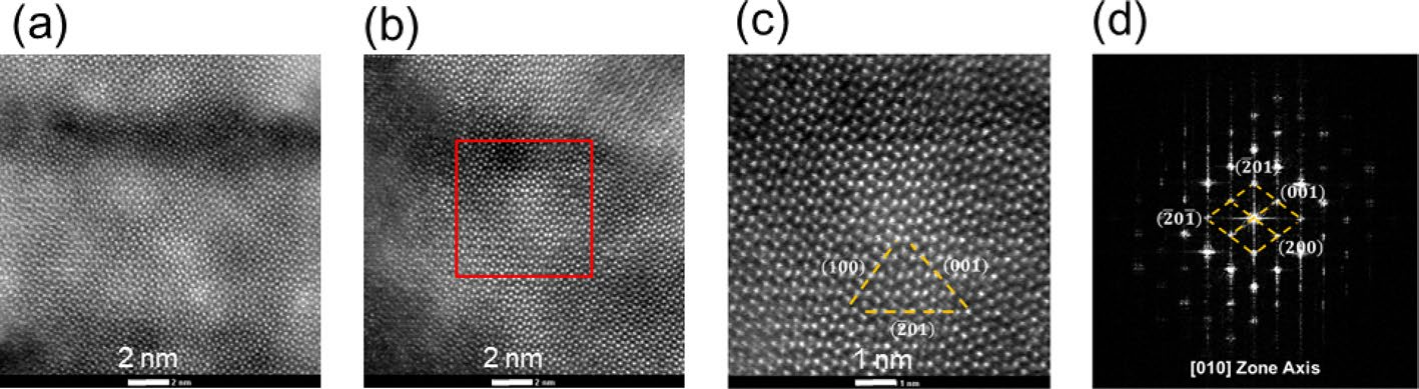
High resolution STEM images obtained from the MOCVD grown β-(Al_*x*_Ga_1-*x*_)_2_O_3_ thin films grown with different TMAl molar flow rates of **a** 1.12 × 10^–5^ mol/min and **b** 2.24 × 10^–5^ mol/min. **c** HAADF STEM image from the rectangular area (red) in (**b**), and **d** the corresponding Fast Fourier Transform (FFT) analysis of (**c**)

To investigate the microstructure, (Al_*x*_Ga_1-*x*_)_2_O_3_ epilayers deposited with high molar flow rates of TMAl were examined by STEM. Typical low-magnification STEM micrographs are shown in Fig. 6. The STEM images were obtained from the β-(Al_*x*_Ga_1-*x*_)_2_O_3_ deposited by MOCVD with 2.24 × 10^–5^ mol/min, (Fig. 6a) and 3.36 × 10^–5^ mol/min (Fig. 6b) TMAl molar flow rates. The corresponding microstructure was composed primarily of β-(Al_*x*_Ga_1-*x*_)_2_O_3_. The dark flake-like β-(Al_*x*_Ga_1-*x*_)_2_O_3_ could often be observed in the epitaxial layers (see Fig. 6a). The observed flakes likely originate from local structural modifications caused by the Al addition, progressively merging into coalesced structures and eventually forming a dark band-like β-(Al_*x*_Ga_1-*x*_)_2_O_3_ with the rise in Al concentration (Fig. 6b). There is a need to explain the cause of the striking features of the dark regions and how they influence the deposition of β-(Al_*x*_Ga_1-*x*_)_2_O_3_. To clarify the mechanism of this phenomenon, the dark regions were probed using atomic resolution STEM images (Fig. 7).

The dark regions shown in Fig. 7 are confined to the nanometer scale and correspond to Al-rich β-(Al_*x*_Ga_1-*x*_)_2_O_3_ compositional domain. The epitaxial layers display a uniform cross section without extended defects. The dark contrast reflects regions with higher Al content relative to the surrounding, owing to the atomic number (Z) sensitivity of HAADF STEM imaging. In HAADF-STEM, incoherent imaging is achieved using electrons elastically scattered by atoms, primarily through thermal diffuse scattering (TDS). The regions with Z exhibit greater contrast, typically scaling with the square of the atomic number. As a result, Ga (Z = 31) stands out more brightly compared to Al (Z = 13), so areas rich in Al appear darker relative to Ga-rich regions in these images.

Figure 7c presents an atomic-resolution HAADF-STEM image of the ($\overline{2 }$01)-oriented β-(Al_*x*_Ga_1-*x*_)_2_O_3_ thin film, obtained along the [010] zone axis. This is a magnified view of the area highlighted in Fig. 7b, revealing the detailed characterization of atomic structure in the dark region. The corresponding fast Fourier transform (FFT) analysis is shown in Fig. 7d. The orientation relationship of the β-(Al_*x*_Ga_1-*x*_)_2_O_3_ layer with the c-plane sapphire substrate was determined to be [010]_β-Ga2O3_//[10̅10]_Al2O3_ and ($\overline{2 }$01)_β-Ga2O3_//(0001)_Al2O3_. It is important to note that the aluminium composition in the β-(Al_*x*_Ga_1-*x*_)_2_O_3_ films lies within a moderate range. STEM analysis confirmed the epitaxial relationship within a single localized region, revealing coherent alignment between the β-(Al_*x*_Ga_1-*x*_)_2_O_3_ layer and the underlying substrate at that specific site. However, with increasing aluminum concentration, larger lattice mismatch and strain relaxation may introduce greater distortions in the in-plane orientation, potentially affecting the structural uniformity of the film as a whole [50].

The morphology of the Al-rich (dark) and Ga-rich (bright) β-(Al_*x*_Ga_1-*x*_)_2_O_3_ regions suggests that their formation is governed by a thermally driven diffusional precipitation mechanism, which serves to relax lattice strain energy. This relaxation process is accompanied by the formation of nanoscale twins and stacking faults. The β-(Al_*x*_Ga_1-*x*_)_2_O_3_ layer exhibited layer-by-layer growth along the ($\overline{2 }$01)_β-Ga2O3_//(0001)_Al2O3_ orientation. These different solute atoms are segregated around the epitaxial interfaces due to the high lattice strain energy. It should be noted that the Al incorporation is not entirely homogeneous throughout the film thickness and shows depth-dependent variation. Therefore, the reported Al composition represents an effective average Al content of the films. Nevertheless, it remains useful for correlating the overall structural and growth trends with TMAl flow variation.

**Fig. 8:**
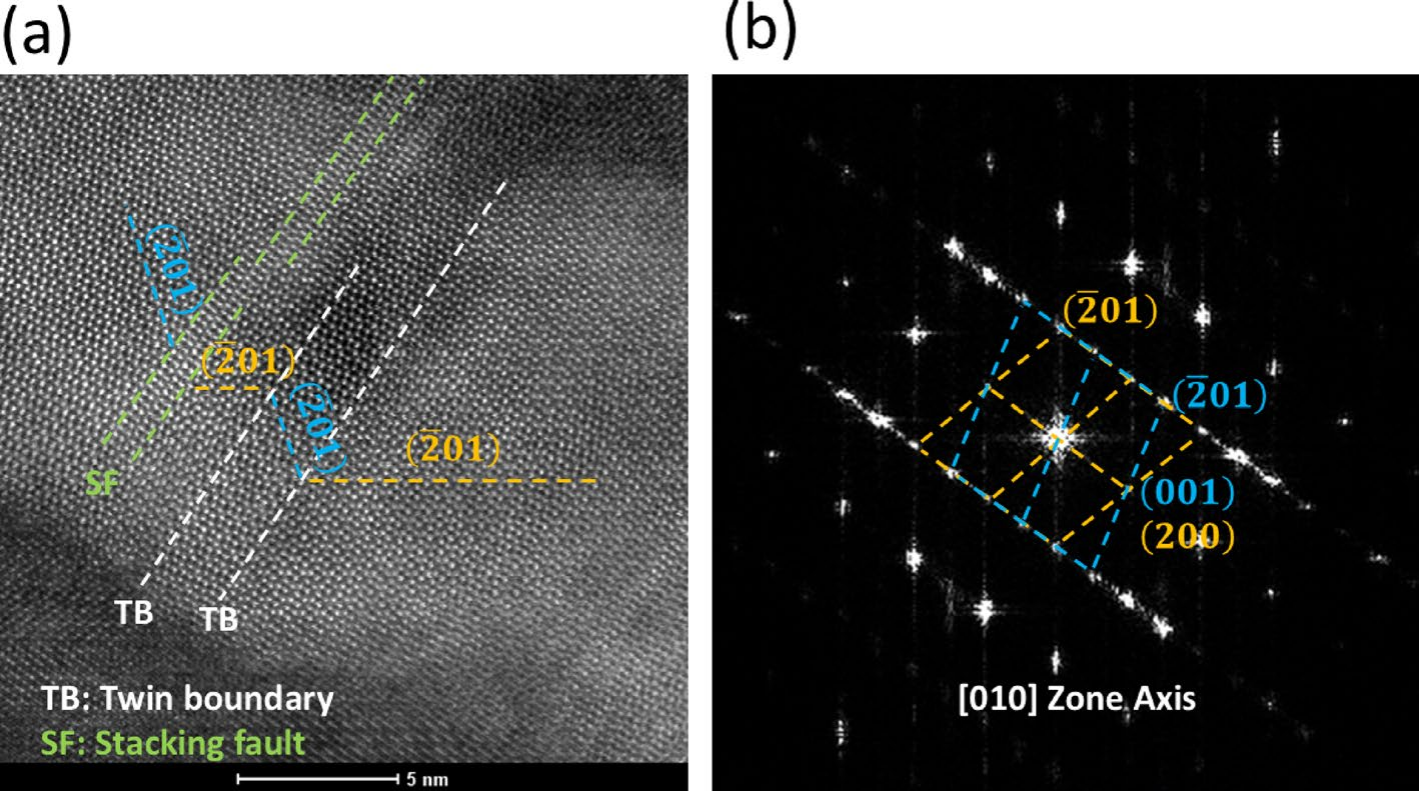
**a** Nano-twins and stacking faults in the β-(Al_*x*_Ga_1-*x*_)_2_O_3_ epitaxial layer deposited with high molar flow rate of TMAl, and **b** the associated FFT analysis of (**a**)

Figure 8a reveals nanoscale twins and stacking faults forming a coalescence band structure in the β-(Al_*x*_Ga_1-*x*_)_2_O_3_ layer deposited under a high molar flow rate of TMAl. These defects were frequently seen along the [010] β-Ga_2_O_3_ zone axis, as presented in Fig. 8b, and their formation is consistent with a layer-by-layer growth mechanism. Additionally, Fig. 8b displays two diffraction patterns taken along the same [010] zone axis, identifying the nano-twins with coincidence twin planes of (100) and (001), respectively.

**Fig. 9:**
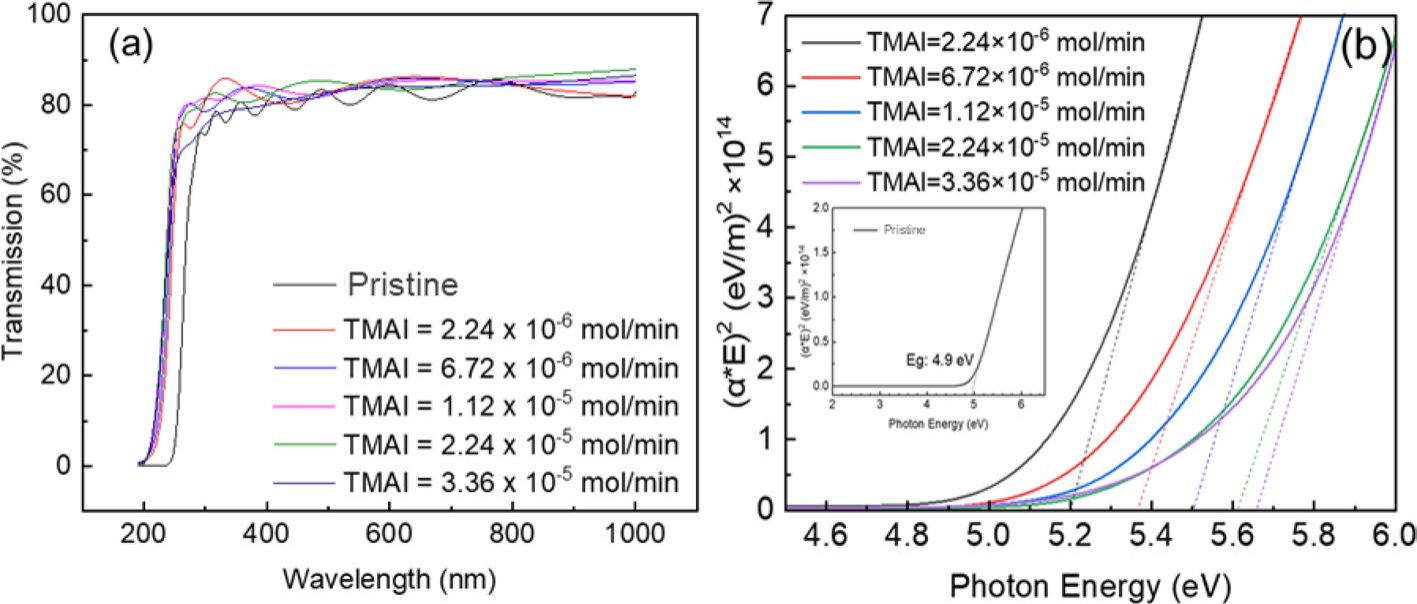
**a** Transmittance versus wavelength, and **b** Tauc plots with their linear fits for the (Al_*x*_Ga_1-*x*_)_2_O_3_ epilayers deposited with varied molar flow rates of TMAl

Figure 9a presents the measured optical transmittance of the investigated samples as a function of wavelength. All samples exhibited transmittance values exceeding 80% for wavelengths above 300 nm. For shorter wavelengths the transmittance drops and reaches 0 below 250 nm for the sample with a pristine β-Ga_2_O_3_ epilayer and below 200 nm for samples with (Al_*x*_Ga_1-*x*_)_2_O_3_ epilayers. This indicates that the optical bandgaps of (Al_*x*_Ga_1-*x*_)_2_O_3_ are wider than the pristine β–Ga_2_O_3_. To obtain estimates of the optical bandgap (${E}_{\mathrm{g}})$ we used the transmittance data,

6 $$ \alpha^{*} = - \left( \frac{1}{d} \right) \cdot \ln \left( T \right). $$

This expression gives an absorption coefficient ${\alpha }^{*}$, which is an approximation of the intrinsic absorption coefficient $(\alpha$) of the epilayers (${\alpha }^{*}\approx \alpha )$. The approximative absorption coefficient is dependent only on $T$ and the epilayer thickness $(d)$. Thus, from the transmittance measurements and the thicknesses from the SEM study we could obtain ${\alpha }^{*}$ as dependent on photon energy ($E$). To estimate the optical bandgaps ${E}_{\mathrm{g}}$ we choose to use use the well-known Tauc method [51] assuming direct transitions. That is, we use the expression,

7 $$ \left( {\alpha^{*} E} \right)^{2} \propto E, $$

where the estimated ${E}_{\mathrm{g}}$ is found from a linear extrapolation in the plot. The resulting Tauc plots and linearizations are presented in Fig. 9b and Table 3, showing that the estimated ${E}_{\mathrm{g}}$ increases from 4.92 eV for the pristine β-Ga_2_O_3_ epilayer to 5.66 eV for epilayers deposited by molar flow rates of TMAl:2.24 × 10^–5^ and 3.36 × 10^–5^ mol/min. Even though the bandgap values are derived from an approximative absorption coefficient and that care should be taken using Tauc linearization we can conclude that we see a monotonic increase of ${E}_{\mathrm{g}}$ with Al incorporation confirming the optical tunability of the (Al_*x*_Ga_1-*x*_)_2_O_3_.

**Table 3:** Estimated optical bandgaps of the pristine β-Ga_2_O_3_ and (Al_x_Ga_1-x_)_2_O_3_ epilayers

TMAl molar flow rate (mol/min)	Optical Energy bandgap, ${E}_{\mathrm{g}}$ (eV)	Calculated Energy bandgap by Al content, (eV)
0 (pristine)	4.92	4.92
2.24 × 10^–6^	5.20	5.03
6.72 × 10^–6^	5.37	5.21
1.12 × 10^–5^	5.50	5.39
2.24 × 10^–5^	5.61	5.64
3.36 × 10^–5^	5.66	5.82

The observed trends are in agreement with earlier reports on ultra-wide bandgap semiconductors [52–54]. In general, a higher number of interference maxima and minima in a transmission spectrum indicates a larger optical thickness. However, in the (Al_x_Ga_1−x_)_2_O_3_ alloy, the refractive index decreases with increasing Al content [55]. As a result, the fringe count alone cannot reliably indicate the physical thickness of the films. Thus, SEM cross-sections provide a direct and more accurate measurement of the actual thickness. The demonstrated capability for bandgap engineering further underscores its suitability for deep-UV applications and operation in high electric field environments.

The ${E}_{\mathrm{g}}$ of (Al_x_Ga_1−x_)_2_O_3_ can be expressed by the following equation [56, 57]:

8 $$ E_{g } {\text{ (Al}}_{x} {\mathrm{Ga}}_{1 - x} )_{2} {\mathrm{O}}_{3} { = }E_{g } {\mathrm{(Ga}}_{2} {\mathrm{O}}_{3} {)}{\text{.(1 - x) + }}E_{g } {\mathrm{(Al}}_{2} {\mathrm{O}}_{3} {)}{\text{.x - }}C_{0 } {\text{. x }}{\text{. (1 - x)}} $$

where ${E}_{g }({Ga}_{2}{O}_{3})$ and ${E}_{g }({Al}_{2}{O}_{3})$ correspond to the bandgaps of the two binary end members, Ga_2_O_3_ and Al_2_O_3_, respectively. The parameter ${C}_{0}$, referred to as the bowing parameter, quantifies the degree of deviation from linear relationship with the bandgaps of two end-member. A nonzero bowing parameter arises due to differences in atomic sizes, electronegativities, and the electronic structures of the constituent materials, which lead to non-linear changes in the valence and conduction band edges. The value of the C_0_ was determined to be 1.7, consistent with earlier reports [58, 59]. Table 2 presents the bandgap values estimated using the extracted bowing parameter. These values show good agreement with experimental results, except at higher Al concentrations (> 30%), where pronounced lattice disorder leads to noticeable deviations.

Since Al incorporation directly influences both the crystallinity and the deposition rate of the (Al_*x*_Ga_1-*x*_)_2_O_3_ epilayers, as evidenced by the crystal structure and increased thickness with higher TMAl molar flow rate—it becomes imperative to quantify the Al composition reliably. Based on the previous XRD results, it can be inferred that the larger Ga atoms in the (Al_*x*_Ga_1-*x*_)_2_O_3_ epilayers have been substituted by smaller Al atoms. However, determining the value of *x* in (Al_*x*_Ga_1-*x*_)_2_O_3_ reliably through the optical bandgap measurements still remains unclear. However, the obtained values of optical bandgap confirm a strong correlation between the TMAl precursor molar flow rate during the growth and Al incorporation, which in turn governs the structural evolution and growth kinetics of the (Al_*x*_Ga_1-*x*_)_2_O_3_ epilayers.

**Fig. 10:**
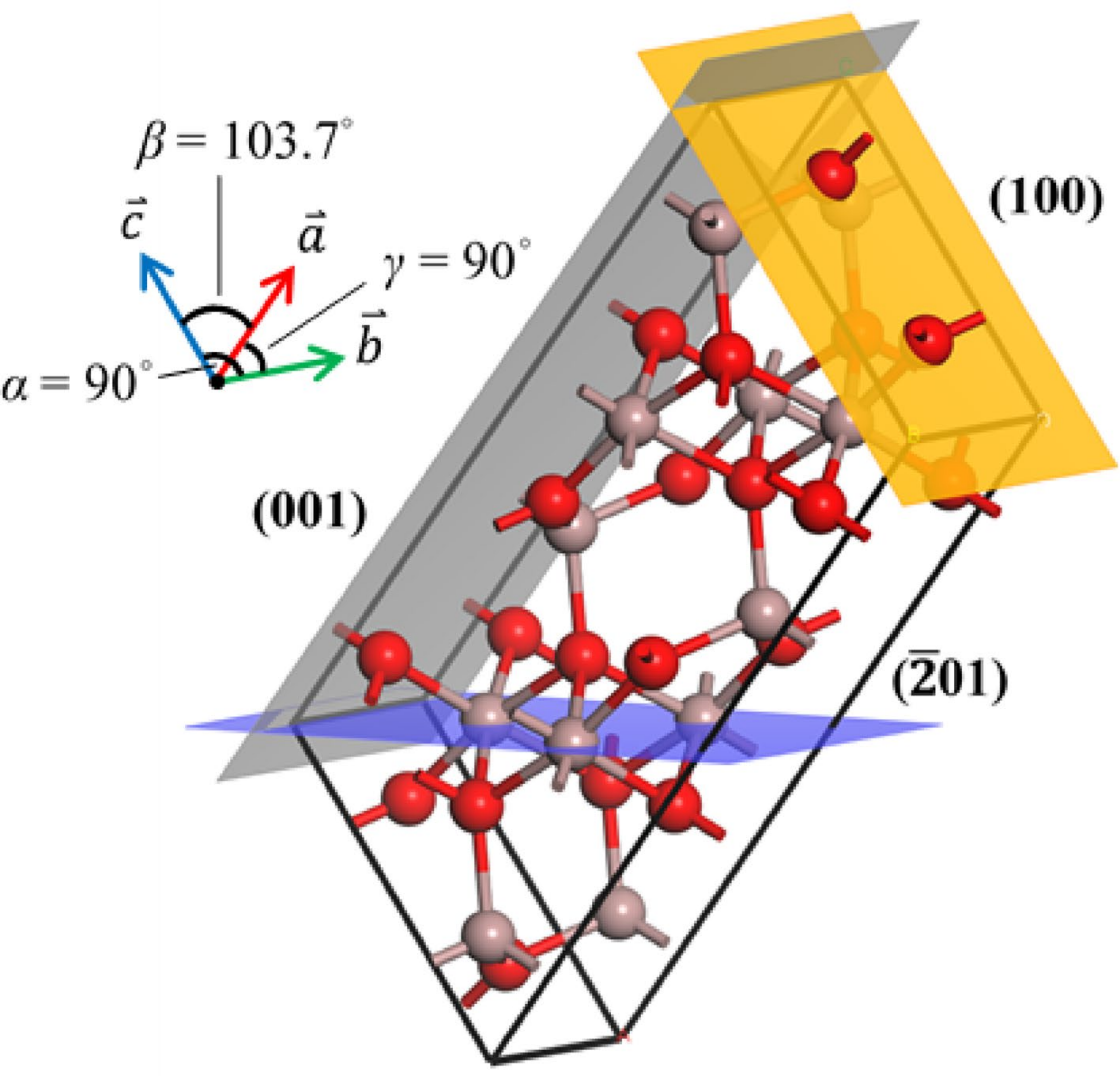
Crystallographic model of monoclinic β-Ga_2_O_3_ showing the (100), (001), and ($\overline{2 }$01) planes within the unit cell. The lattice vectors *a*, *b*, and *c* are illustrated together with the monoclinic angle *β* = 103.7° between the *a* and *c* axes. The (100) plane, highlighted in orange, corresponds to the surface considered in the surface-energy calculations and morphology analysis. Oxygen and gallium atoms are represented by red and brown spheres, respectively

Furthermore, to elucidate the growth mechanism about the crystallographic nature of the rectangular platelet morphology observed in the SEM image (see Fig. 4f), we conducted a detailed structural analysis based on atomic-resolution HAADF-STEM images (see Figs. 7 and 8). The STEM cross-sectional image in Fig. 7c was acquired along the [010] zone axis, as validated by the corresponding FFT pattern in Fig. 7d, which reveals a lattice cross-section in which the electron beam is oriented parallel to the *b*-axis. Figure 10 illustrates that the electron beam is parallel to the [010] crystallographic direction, and the image reveals the atomic structure within the (100) and (001) planes. This indicates a platelet structure with a surface normal along the [100] direction or the *a*-axis. Based on the atomic column arrangements, it is evident that the crystal predominantly extends within the (100) plane along the [010] and [001] directions or the *b*-axis and the *c*-axis. As such, the platelet surface normal corresponds to the [100] direction, and the observed SEM morphology in Fig. 4f is attributed to the (100) surface of β-(Al_*x*_Ga_1-*x*_)_2_O_3_. Specifically, the [010] direction is known to be the densest atomic packing direction in β-Ga_2_O_3_ and is often associated with favorable growth and charge transport properties [60]. In crystals with layered or anisotropic stacking sequences, stacking faults along the [001] direction represent planar defects characterized by local disruptions in the regular stacking order of atomic planes through omissions, shifts, or misplacements of atomic layers (see Fig. 10). These faults frequently occur on crystallographic slip planes, where weaker bonding, less stable coordination environments, or large interlayer spacings increase the likelihood of atomic misalignment. Anisotropy in bonding directions may further facilitate the occurrence of such faults. During crystal growth, the formation of stacking faults can serve as a mechanism for strain relaxation, helping to reduce internal stress and lower the system’s total energy, thereby stabilizing local regions of the crystal. The presence of stacking faults also indicates that the crystal exhibits limited capability to maintain an ordered stacking sequence along the *c*-axis. Uncontrolled propagation of stacking faults hinders both high-quality growth and the growth rate along the affected crystallographic directions. Thus, the role of stacking faults in crystal growth is intrinsically dual: they can be beneficial for stress relief, yet detrimental to structural coherence and crystalline perfection, particularly when they propagate along the *c*-axis growth directions.

Based on this structural analysis, we conclude that the rectangular platelet morphology observed in Fig. 4f originates from lateral growth on the (100) surface of β-(Al_*x*_Ga_1-*x*_)_2_O_3_. Moreover, the prominence of this rectangular morphology increases with Al content, indicating that Al incorporation enhances the lateral growth tendency along the (100) facet. To further understand this growth behavior from a thermodynamic perspective, the surface energetics were evaluated based on chemical potential analysis. The chemical potential of Ga_2_O_3_ is determined from the chemical potentials of Ga and O, denoted as ${\mu}_{Ga}$ and ${\mu}_{O}$, respectively. According to thermodynamic constraints, the allowable ranges of ${\mu}_{Ga}$ and ${\mu}_{O}$ are given by,

9 $$ \mu_{{{\mathrm{Ga}}}}^{{{\mathrm{bulk}}}} + \Delta H^{f} \left[ {Ga_{2} O_{3} } \right] \le \mu_{{{\mathrm{Ga}}}} \le \mu_{{{\mathrm{Ga}}}}^{{{\mathrm{bulk}}}} $$

and

10 $$ \frac{1}{2}\mu_{{{\mathrm{O}}_{2} }}^{{{\mathrm{gas}}}} { + }\Delta H^{f} \left[ {{\mathrm{Ga}}_{{2}} {\mathrm{O}}_{{3}} } \right] \le { }\mu_{{\mathrm{O}}} { } \le { }\frac{1}{2}\mu_{{{\mathrm{O}}_{2} }}^{{{\mathrm{gas}}}} $$

These ranges are bounded by $\mu_{{{\mathrm{Ga}}}} { } \le { }\mu_{{{\mathrm{Ga}}}}^{{{\mathrm{bulk}}}}$, $\mu_{{\mathrm{O}}} { } \le { }\frac{1}{2}\mu_{{{\mathrm{O}}_{2} }}^{{{\mathrm{gas}}}}$, and ${2}\mu_{{{\mathrm{Ga}}}} { + 3}\mu_{{\mathrm{O}}} { = }\mu_{{{\mathrm{Ga}}_{{2}} {\mathrm{O}}_{{3}} }}^{{{\mathrm{bulk}}}}$. Equation (10) can be simplified to

11 $$ \Delta H^{f} \left[ {{\mathrm{Ga}}_{{2}} {\mathrm{O}}_{{3}} } \right]{ } \le { }\mu_{{\mathrm{O}}} { } - { }\frac{1}{2}\mu_{{{\mathrm{O}}_{2} }}^{{{\mathrm{gas}}}} { } \le 0 $$

or

12 $$ \Delta H^{f} \left[ {{\mathrm{Ga}}_{{2}} {\mathrm{O}}_{{3}} } \right]{ } \le { }\Delta \mu_{{\mathrm{O}}} { } \le 0 $$

The formation enthalpy of bulk Ga_2_O_3_ is defined as:

13 $$ \Delta H^{f} \left[ {{\mathrm{Ga}}_{{2}} {\mathrm{O}}_{{3}} } \right]{ = }\mu_{{{\mathrm{Ga}}_{{2}} {\mathrm{O}}_{{3}} }}^{{{\mathrm{bulk}}}} { } - 2\mu_{{{\mathrm{Ga}}}}^{{{\mathrm{bulk}}}} { } - \frac{3}{2}\mu_{{{\mathrm{O}}_{2} }}^{{{\mathrm{gas}}}} $$

Similarly, the allowable range for $\mu_{{{\mathrm{Al}}}}$ is:

14 $$ \mu_{{{\mathrm{Al}}}}^{{{\mathrm{bulk}}}} { + }\Delta H^{f} \left[ {{\mathrm{Al}}_{{2}} {\mathrm{O}}_{{3}} } \right]{ } \le { }\mu_{{{\mathrm{Al}}}} { } \le { }\mu_{{{\mathrm{Al}}}}^{{{\mathrm{bulk}}}} $$

This range is bounded by $\mu_{{{\mathrm{Al}}}} { } \le { }\mu_{{{\mathrm{Al}}}}^{{{\mathrm{bulk}}}} ,{ }\mu_{{\mathrm{O}}} { } \le { }\frac{1}{2}\mu_{{{\mathrm{O}}_{2} }}^{{{\mathrm{gas}}}} ,{\text{ and }} {2}\mu_{{{\mathrm{Al}}}} { + 3}\mu_{{\mathrm{O}}} { = }\mu_{{{\mathrm{Al}}_{{2}} {\mathrm{O}}_{{3}} }}^{{{\mathrm{bulk}}}} .$ Equation (14) can be simplified to

15 $$ \Delta H^{f} \left[ {{\mathrm{Al}}_{{2}} {\mathrm{O}}_{{3}} } \right]{ } \le { }\mu_{{{\mathrm{Al}}}} { } - { }\mu_{{{\mathrm{Al}}}}^{{{\mathrm{bulk}}}} { } \le 0 $$

or

16 $$ \Delta H^{f} \left[ {{\mathrm{Al}}_{{2}} {\mathrm{O}}_{{3}} } \right]{ } \le { }\Delta \mu_{{{\mathrm{Al}}}} { } \le 0 $$

The formation enthalpy of bulk Al_2_O_3_ is defined as:

17 $$ \Delta H^{f} \left[ {{\mathrm{Al}}_{{2}} {\mathrm{O}}_{{3}} } \right]{ = }\mu_{{{\mathrm{Al}}_{{2}} {\mathrm{O}}_{{3}} }}^{{{\mathrm{bulk}}}} { } - 2\mu_{{{\mathrm{Al}}}}^{{{\mathrm{bulk}}}} { } - \frac{3}{2}\mu_{{{\mathrm{O}}_{2} }}^{{{\mathrm{gas}}}} $$

**Fig. 11:**
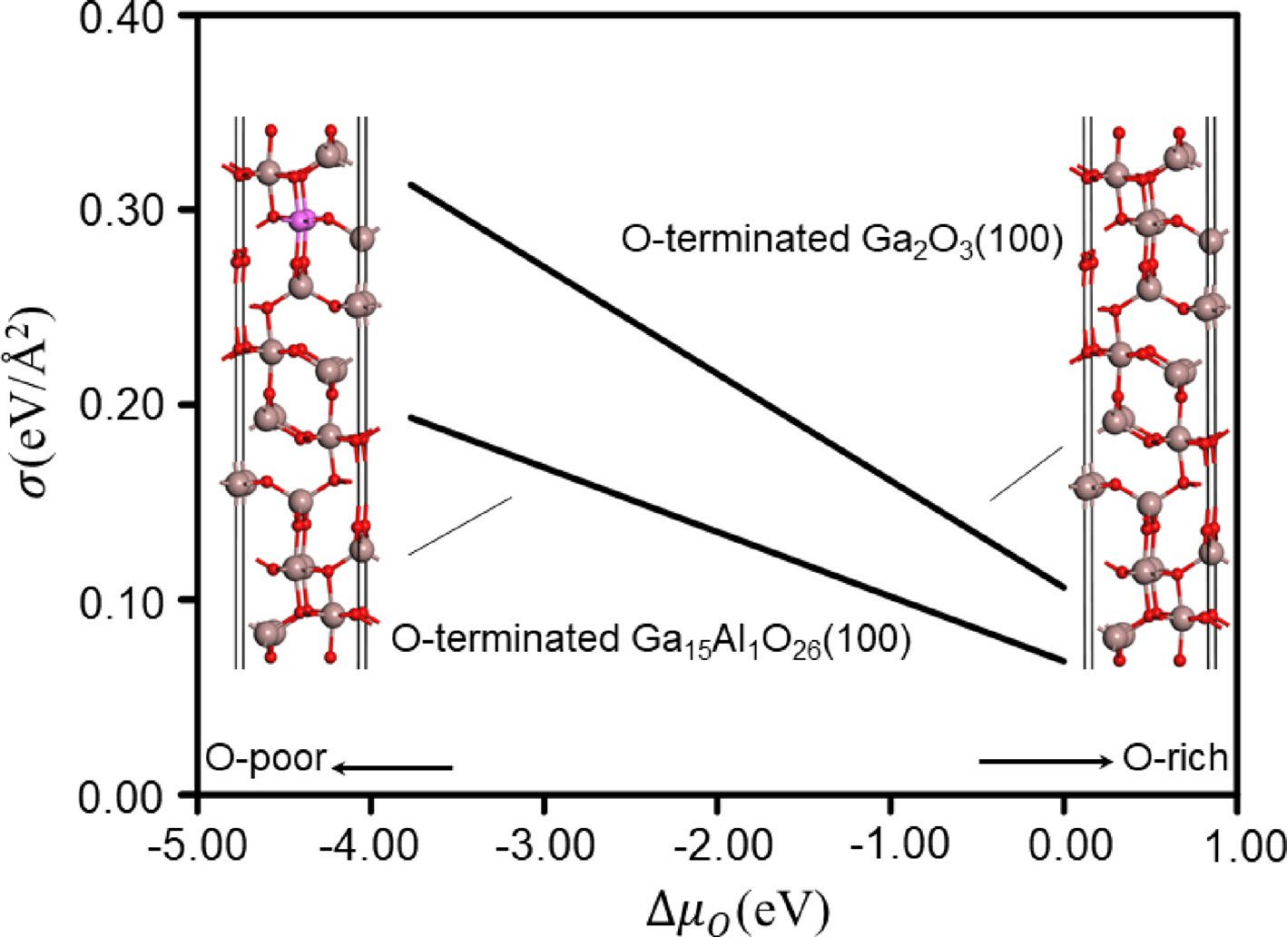
Surface energy variation of O-terminated Ga_2_O_3_(100) and Ga_15_Al_1_O_26_(100) surfaces as a function of oxygen chemical potential, showing enhanced stability of the Al-substituted surface under O-rich conditions. The atomic models represent the relaxed surface slab geometries of Ga_15_Al_1_O_26_ (left, with Al in pink) and β-Ga_2_O_3_(100) (right, with Ga in Brown and O in red), both terminated by oxygen

The surface energy of the Ga_2_O_3_(100) surface is determined by the following:

18 $$ \sigma = {{\left( {E_{{{\mathrm{slab}}}} - \mathop \sum \limits_{i} n_{i} \mu_{i} } \right)} \mathord{\left/ {\vphantom {{\left( {E_{{{\mathrm{slab}}}} - \mathop \sum \limits_{i} n_{i} \mu_{i} } \right)} {2{\mathrm{A}}}}} \right. \kern-0pt} {2{\mathrm{A}}}}, $$

where σ is the surface energy, ${n}_{i}$ and ${\mu}_{i}$ are the number,* E*_slab_ is the total energy of the vacuum slab model and chemical potential of each species, respectively, and *A* is the surface area. The values of ${\mu}_{Ga}^{bulk}$, ${\mu}_{{\mathrm{O}}_{2}}^{gas}$, ${\mu}_{{Ga}_{2}{O}_{3}}^{bulk}$, ${\mu}_{Al}^{bulk}$, and ${\mu}_{{Al}_{2}{O}_{3}}^{bulk}$ are obtained from the total per atom energies of bulk Ga (space group: 64 *Cmca*), O_2_ gas (space group: 123 *P*4/*mmm*), bulk Ga_2_O_3_ (space group: 12 C2/m), bulk Al (space group: 225 *Fm* $\overline{3 }$ *m*), and bulk Al_2_O_3_ (space group: 167 *R* $\overline{3 }$ *c*), respectively. Our calculated formation enthalpies of $\Delta {H}^{f}\left[{Ga}_{2}{O}_{3}\right]$ and $\Delta {H}^{f}\left[{Al}_{2}{O}_{3}\right]$ are $-$ 11.30 eV and $-$ 17.18 eV, which are consistent with reported values: $-$ 10.40 eV for Ga_2_O_3_ [61] and $-$ 17.22 eV for Al_2_O_3_ [62]. The calculated surface energies of the O-terminated Ga_2_O_3_(100) and Ga_15_Al_1_O_26_(100) surfaces versus oxygen chemical potential are shown in Fig. 11. For both surfaces, the surface energies decreased under oxygen rich conditions, indicating enhanced thermodynamic stability. The computed values range from 0.106 to 0.313 eV/Å^2^ for Ga_2_O_3_(100) surface and 0.068–0.193 eV/Å^2^ for Ga_15_Al_1_O_26_(100), in good agreement with prior theoretical data, such as the 0.105 eV/Å^2^ value reported by Bermudez [63]. The Ref. [63] also notes that β-Ga_2_O_3_ (100) surface being most stable among the major low-index planes, explaining the preferential exposure of the (100) surface during equilibrium crystal growth, which aligns with the experimentally observed rectangular platelet morphology dominated by (100) facets. Notably, the Al-substituted Ga_15_Al_1_O_26_ remains consistently lower surface energies than that of pure Ga_2_O_3_ across the entire range, reaching a minimum value of 0.068 eV/Å^2^ under O-rich conditions. In the context of crystal growth theory, a facet with a lower surface energy tends to exhibit a reduced normal growth rate, which promotes their lateral expansion while higher-energy facets grow out and vanish more quickly. This indicates that Al incorporation effectively stabilizes the O-terminated (100) surface, promoting lateral growth along this plane and thereby validating the rectangular platelet morphology observed experimentally in Fig. 4f. This mechanism is general and has been observed in other oxide systems, underscoring its applicability here. This morphological transition is entirely consistent with the DFT-predicted stabilization of the (100) facet, providing a rare direct correspondence between atomistic-level thermodynamic calculations and macroscopic growth outcomes. The structural models shown alongside the plot in Fig. 11 illustrate the relaxed slab geometries used for the surface energy calculations, with the left structure representing the Ga_15_Al_1_O_26_ slab (highlighting the substitutional Al atom in pink), and the right structure representing the pure β-Ga_2_O_3_(100) slab.

While earlier theoretical studies mainly focused on pristine β–Ga_2_O_3_ surfaces, the present work systematically evaluates the effect of partial Al substitution on the O-terminated β–Ga_2_O_3_(100) surface over the full oxygen chemical potential range relevant to MOCVD growth. To our knowledge, we present the first set of DFT-calculated surface energies for doped β–Ga_2_O_3_ surfaces, providing a direct thermodynamic basis for evaluating the impact of Al incorporation on morphology evolution. This study reveals Al substitution significantly reduces the surface energy of the O-terminated (100) plane, lowering it from 0.313 to 0.068 eV/Å^2^, with the magnitude of the reduction persisting across the entire O-poor to O-rich chemical potential range. This doping-induced stabilization provides a direct thermodynamic explanation for the morphology evolution and establishes a quantitative framework for understanding morphology control through Al incorporation.

Segregation energy calculations for β-Ga_2_O_3_ Σ5(201)[010] grain boundary reveal that substituting a Ga atom at the Σ5(201)[010] boundary with an Al atom reduces the total energy of the system, yielding a segregation energy of − 3.65 eV. This negative value demonstrates that Al atoms are thermodynamically stabilized at the grain boundary and tend to accumulate there rather than remaining uniformly distributed in the bulk. The stabilization arises primarily from local structural relaxation and charge redistribution in the boundary region. Because the Al–O bond (≈ 1.92 Å) is shorter than the Ga–O bond (≈ 1.97 Å), Al substitution locally reduces tensile strain and bond-angle distortion, creating a more compact and symmetric boundary configuration. Moreover, the slightly higher electronegativity of Al enhances electron localization on O atoms, thereby lowering the local Coulombic energy and smoothing the potential landscape across the interface. From an electronic-structure standpoint, the shorter Al–O bond and higher electronegativity of Al are expected to induce local charge redistribution near the interface, which could potentially lower the valence-band edge and suppress interfacial defect states. The combined effects of lattice relaxation and electronic stabilization provide a clear thermodynamic basis for the Al-driven lateral growth behavior observed experimentally. As Al atoms segregate preferentially to the (100)-related boundaries, the surface energy and interface energy of these planes decrease, promoting lateral extension of low-energy facets and leading to the rectangular platelet morphology characteristic of high-Al films.

## Conclusions

(Al_x_Ga_1−x_)_2_O_3_ epilayers deposited via MOCVD with different molar flow rates of TMAl (2.24 × 10^–6^ to 3.36 × 10^–5^ mol/min) were systematically examined to assess their structural and electronic properties. XRD analysis confirmed progressive Al incorporation into the β–Ga_2_O_3_ lattice, resulting in peak shifts toward higher diffraction angles and a systematic reduction in lattice parameters. The Al content in (Al_x_Ga_1−x_)_2_O_3_ epilayers was evaluated as 0%, 6.0%, 14%, 21%, 30%, and 36%, for pristine β-Ga_2_O_3_, and (Al_x_Ga_1−x_)_2_O_3_ films grown with 2.24 × 10^–6^ to 3.36 × 10^–5^ mol/min of TMAl, respectively. Good agreement was found by XPS analysis for low-Al content samples, while notably higher Al content was evaluated for films grown with TMAl flows of 2.24 × 10^–5^ and 3.36 × 10^–5^. Cross-sectional SEM analysis revealed thickening of the film from 153 nm (undoped) to 278 nm (3.36 × 10^–5^ mol/min), indicating enhanced growth kinetics with higher TMAl molar flow rates. Optical absorption studies estimated the bandgap of (Al_x_Ga_1−x_)_2_O_3_ to be tunable from 4.92 to 5.66 eV with Al content varying from 0 to 0.36. Thermodynamic analysis revealed that Al incorporation lowers the surface energy of the O-terminated (100) facet under O-rich conditions, thereby stabilizing this surface and promoting lateral growth. This stabilization provides a direct explanation for the experimentally observed rectangular platelet morphology. Overall, our results provide detailed insights towards the structure–property relationships of (AlGa_1-x_)_2_O_3_ epilayers and underscore their strong capability for use in deep-ultraviolet optoelectronics and high-power electronics that rely on ultrawide-bandgap semiconductors.

## Data Availability

The datasets generated during and/or analyzed during the current study are available from the corresponding author on reasonable request.
